# Editorial: Neural signals acquisition and intelligent analysis

**DOI:** 10.3389/fnins.2023.1251280

**Published:** 2023-07-20

**Authors:** Gwanggil Jeon, Xiaomin Yang, Yin Tian, Yu Pang

**Affiliations:** ^1^Department of Embedded Systems Engineering, College of Information Technology, Incheon National University, Incheon, Republic of Korea; ^2^College of Electronics and Information Engineering, Sichuan University, Chengdu, China; ^3^Department of Biomedical Engineering, Chongqing University of Posts and Telecommunications, Chongqing, China; ^4^School of Communication and Information Engineering, Chongqing University of Posts and Telecommunications, Chongqing, China

**Keywords:** signal, neural network, intelligent system, EEG, medical information

Neural signals include physiological and pathological information, which requires psychological, biological, cognitive neuroscience, and clinical medicine information to efficiently analyze them. So far, it has been possible to record and analyze neural signals using EEG, brain magnetometer, functional magnetic resonance imaging, and computed tomography. On the other hand, artificial intelligence technology is becoming increasingly popular, and a neuroscience technology based on deep learning has recently been announced. This Research Topic aims to apply research on AI-based pattern recognition and signal processing to neuroscience. Interactions between these signal processing fields and the medical field will aid in the diagnosis, monitoring and treatment of neurological disorders. A total of 9 papers were published in this Research Topic.

EEG is used to measure the brain's electrical activity in a non-invasive brain-machine interface. Since the EEG signal is a non-linear and non-static signal, interpretation is difficult, but improved results have been obtained with the development of deep learning technology. In the contribution by de Oliveira and Rodrigues “*Empirical comparison of deep learning methods for EEG decoding*,” the authors present two deep learning-based decoder implementations and compare their results with other state-of-the-art deep learning methods. The first method uses LSTM recurrent neural networks, and the second method combines EEGNet with LSTM. The results of this work could be important for new research and development as well as EEG-based BMI systems that can exploit the high precision of neural decoders.

In the contribution by Kuroda et al. “*Detection of astrocytic slow oscillatory activity and response to seizurogenic compounds using planar microelectrode array*,” the authors studied the measurement of the spontaneous electrical activity of astrocytes alone using MEA. It was revealed that MEA measurement focused on the low frequency band could be used as one of the methods to evaluate drug response *in vitro*. The authors established nine parameters to evaluate astrocyte activity and evaluated five paroxysmal drug responses in human primary astrocytes and human iPSC-derived astrocytes. Astrocytes showed the most significant dose-dependent changes with pilocarpine. Principal component analysis using these parameter sets isolated the drug response to each seizure-inducing compound.

A multi-frequency steady-state visual evoked potential stimulation and decoding method enables the representation of various visual objects in a brain-computer interface. However, unlike single-frequency SSVEP, multi-frequency SSVEP is difficult to use. One of the main reasons is that it is difficult to define an effective set of frequencies for an interface due to duplication of input options. In the contribution by Mu et al. “*Frequency set selection for multi-frequency steady-state visual evoked potential-based brain-computer interfaces*” provides guidelines for frequency set selection in multi-frequency SSVEP. The proposed method showed a significant improvement in BCI performance (decoding accuracy) compared to the existing method. Both hypotheses were verified experimentally.

Medical information represented by MRI and PET has contributed to the development of intelligent diagnosis of Alzheimer's disease and multimodal medical imaging. This improves the existing multi-medical image fusion method based on sparse expression in terms of energy and contrast. In the contribution by Zhang et al. “*A multimodal fusion method for Alzheimer's disease based on DCT convolutional sparse representation*,” the authors propose a multimodal convergence algorithm for Alzheimer's disease based on DCT convolutional sparse representation. Extensive experimental results demonstrate that the proposed method has excellent performance in enhancing contrast and maintaining texture and contour information.

Although many studies have been conducted on the characteristics of epileptic electroencephalograms, many studies are still needed. In the contribution by Lu et al. “*Study on characteristic of epileptic multi-electroencephalograph base on Hilbert-Huang transform and brain network dynamics*,” a combination method of multi-channel characteristics in time-frequency and spatial domains was studied to study the characteristics of epileptic EEG signals from the perspective of the whole brain. The two contributions of the proposed study are: First, the signal was converted into a 2D Hilbert Spectrum image reflecting time-frequency characteristics through the Hilbert-Huang Transform. Second, multi-channel signals were converted into brain networks reflecting spatial characteristics by Symbolic Transfer Entropy between different EEG channels. When looking at the experimental results, it was found that it is effective in identifying and predicting epileptic seizures.

Traumatic brain injury, one of the major public health problems in children, leads to the development of attention deficit. Existing studies have shown that structural and functional changes in several brain regions are associated with TBI-related attention deficits in children. In the contribution by Cao et al. “*Abnormal structural and functional network topological properties associated with left prefrontal, parietal, and occipital cortices significantly predict childhood TBI-related attention deficits: a semi-supervised deep learning study*,” the authors developed a method to provide accurate diagnosis by applying deep learning technology to multidimensional and non-linear information. In addition, a semi-supervised autoencoder, a deep learning model, was constructed to investigate the phase change of both structural and functional brain networks in children with TBI and their predictive power for attention deficit after TBI. As a result of the experiment, the proposed model was able to discriminate children with TBI and control groups with an average accuracy of 82.86%.

Epilepsy is the second most common cranial nerve disease after stroke. Seizure prediction is critical to improving patients' quality of life. In the contribution by Zhong et al. “*Epileptic prediction using spatiotemporal information combined with optimal features strategy on EEG*,” the authors constructed an optimal spatiotemporal feature set to predict seizures from multidimensional perspectives including time-frequency, entropy, and brain networks. The proposed method shows strong independence and capacity for large-capacity information, and a two-dimensional feature screening algorithm was performed to remove unnecessary redundant features. As a result of the experiment, the proposed method was able to effectively extract spatiotemporal information of epileptic EEG signals to predict epileptic seizures with high performance.

BAN is a body-oriented network of wireless wearable devices and is a basic technology for telemedicine service. However, when strengthening BAN security, there is an aspect that makes it difficult to improve performance. In the contribution by Bai et al. “*A data security scheme based on EEG characteristics for body area networks*,” a data encryption method based on EEG feature values and LFSR is proposed to solve the data security problem in BAN. To this end, first, based on the wavelet packet conversion method, the characteristics of the human brain wave signal are extracted as MD5 input data to ensure randomness, and then the LFSR stream key generator is adopted. The effectiveness of the proposed security technique was verified through various experimental evaluations.

Segmentation technology in medical imaging is a key technology that helps doctors accurately analyze the volume of brain tissue and lesions, and is important for accurate diagnosis of brain diseases. Existing manual methods are time-consuming, subjective, and difficult to reproduce in segmentation. In the contribution by Wang et al. “*Energy minimization segmentation model based on MRI images*,” the authors present a method for detecting, characterizing, and quantifying brain tissue and lesions using non-invasive imaging techniques. We also address the effect of multiple sclerosis lesions on the segmentation accuracy of MRI. Experimental verification showed that the proposed AR-FCM algorithm better overcomes the problem of low segmentation accuracy of the RFCM algorithm for tissue border voxels.

## Author contributions

GJ, XY, YT, and YP contributed to conception and design of the study. GJ wrote the first draft of the manuscript. All authors contributed to manuscript, read, and approved the submitted version.

